# Supplementary material for: Trends in admission, resource use and outcomes among elderly patients admitted to an intensive care unit in China
Source: PLoS One. 2026 May 15;21(5):e0348768. doi: 10.1371/journal.pone.0348768 (PMC13178899; doi:10.1371/journal.pone.0348768)
Supplement: S1 Table — (DOCX) [file pone.0348768.s001.docx]

**S1 Table.** Number of admissions and ICU beds over the study period.

|  | Year | | | | | | | |  |
| --- | --- | --- | --- | --- | --- | --- | --- | --- | --- |
|  | 2014 | 2015 | 2016 | 2017 | 2018 | 2019 | 2020 | 2021 | P |
| **ICU data** |  |  |  |  |  |  |  |  |  |
| Total ICU beds, n | 79 | 87 | 87 | 87 | 87 | 116 | 116 | 128 |  |
| Total admissions, n | 3542 | 3529 | 3583 | 3637 | 3772 | 5240 | 3689 | 4543 |  |
| 16-64, n(%) | 1936(54.7) | 1978(56.0) | 2022(56.4) | 2065(56.8) | 2174(57.6) | 3155(60.2) | 2191(59.4) | 2714(59.7) | <0.001 |
| 65-79, n(%) | 1138(32.1) | 1097(31.1) | 1086(30.3) | 1065(29.3) | 1138(30.2) | 1575(30.1) | 1159(31.4) | 1418(31.2) | 0.684 |
| ≥80, n(%) | 468(13.2) | 454(12.9) | 475(13.3) | 507(13.9) | 460(12.2) | 510(9.7) | 339(9.2) | 411(9.0) | <0.001 |
| **Hospital data (all admissions)** |  |  |  |  |  |  |  |  |  |
| Total hospital admissions, n | 46801 | 47560 | 50012 | 53408 | 58822 | 71157 | 46900 | 73771 |  |
| Hospital admissions ≥80 yr, n(%) | 2967(6.34) | 3300(6.94) | 3596(7.19) | 3892(7.29) | 4188(7.12) | 4865(6.84) | 2835(6.04) | 4275(5.79) | <0.001 |

*Note:*P for change in proportion of each cohorts over time.
