# Supplementary material for: Trends in admission, resource use and outcomes among elderly patients admitted to an intensive care unit in China
Source: PLoS One. 2026 May 15;21(5):e0348768. doi: 10.1371/journal.pone.0348768 (PMC13178899; doi:10.1371/journal.pone.0348768)
Supplement: S2 Table — (DOCX) [file pone.0348768.s002.docx]

**S2 Table.** Trends in ICU Population Characteristics.

|  | Year | | | | | | | |  |
| --- | --- | --- | --- | --- | --- | --- | --- | --- | --- |
|  | 2014 | 2015 | 2016 | 2017 | 2018 | 2019 | 2020 | 2021 | P |
| 16-64, n | 1936 | 1978 | 2022 | 2065 | 2174 | 3155 | 2191 | 2714 |  |
| 65-79, n | 1138 | 1097 | 1086 | 1065 | 1138 | 1575 | 1159 | 1418 |  |
| ≥80, n | 468 | 454 | 475 | 507 | 460 | 510 | 339 | 411 |  |
| High comorbidity (CCI≥3), n(%) |  |  |  |  |  |  |  |  |  |
| 16-64 | 452(23.3) | 473(23.9) | 433(21.4) | 467(22.6) | 541(24.9) | 599(19.0) | 499(22.8) | 615(22.7) | 0.119 |
| 65-79 | 478(42.0) | 495(45.1) | 443(40.8) | 447(42.0) | 500(43.9) | 614(39.0) | 497(42.9) | 637(44.9) | 0.754 |
| ≥80 | 234(50.0) | 224(49.3) | 212(44.6) | 233(46.0) | 227(49.3) | 242(47.5) | 155(45.7) | 220(53.5) | 0.539 |
| APACHEII, median(IQR) |  |  |  |  |  |  |  |  |  |
| 16-64 | 6(4-10) | 7(5-10) | 7(5-11) | 7(5-11) | 7(5-11) | 7(5-11) | 9(6-15) | 9(6-15) | ＜0.001 |
| 65-79 | 10(8-14) | 11(9-15) | 11(9-15) | 11(8-16) | 11(8-15) | 11(8-14) | 12(9-17) | 12(9-17) | ＜0.001 |
| ≥80 | 14(10-18) | 13(10-18) | 13(10-17) | 14(10-18) | 14(11-18) | 13(10-18) | 14(11-20) | 14(10-19) | 0.026 |

*Note:*P for change in ICU population characteristics over time.
