# Supplementary material for: Trends in admission, resource use and outcomes among elderly patients admitted to an intensive care unit in China
Source: PLoS One. 2026 May 15;21(5):e0348768. doi: 10.1371/journal.pone.0348768 (PMC13178899; doi:10.1371/journal.pone.0348768)
Supplement: S3 Table — (DOCX) [file pone.0348768.s003.docx]

**S3 Table.** Trends in ICU Resource Use.

|  | Year | | | | | | | |  |
| --- | --- | --- | --- | --- | --- | --- | --- | --- | --- |
|  | 2014 | 2015 | 2016 | 2017 | 2018 | 2019 | 2020 | 2021 | P |
| **Daily average TISS-28, median(IQR)** |  |  |  |  |  |  |  |  |  |
| 16-64 | 21.8(17-24.3) | 21(16.5-24) | 22(17.3-24.5) | 22.2(17.5-24.5) | 22.8(18-24.2) | 24(18.6-24) | 24(20-24.5) | 24(19.6-25.6) | ＜0.001 |
| 65-79 | 21(16.5-24) | 20.7(16.5-24) | 21(17.5-24) | 21.6(17.2-24.3) | 21.1(17.2-24) | 22.2(18-24) | 23.1(18.8-24.3) | 22.8(18.3-25.5) | ＜0.001 |
| ≥80 | 20.1(16-24) | 19.5(16-24) | 20.5(16.2-24) | 21.2(17.4-24.3) | 20.6(17-23.7) | 20.5(17.4-23.8) | 21(17.17-24) | 21.3(17.8-24) | 0.02 |
| **Organ Support,n(%)** |  |  |  |  |  |  |  |  |  |
| Invasive ventilation |  |  |  |  |  |  |  |  |  |
| 16-64 | 540(27.9) | 566(28.6) | 594(29.4) | 593(28.7) | 586(27.0) | 778(24.7) | 658(30.0) | 809(29.8) | 0.792 |
| 65-79 | 287(25.2) | 263(24.0) | 302(27.8) | 296(27.8) | 245(21.5) | 320(20.3) | 306(26.4) | 392(27.6) | 0.985 |
| ≥80 | 96(20.5) | 98(21.6) | 95(20.0) | 117(23.1) | 72(15.7) | 88(17.3) | 57(16.8) | 61(14.8) | 0.001 |
| RRT |  |  |  |  |  |  |  |  |  |
| 16-64 | 18(0.9) | 26(1.3) | 33(1.6) | 28(1.4) | 48(2.2) | 54(1.7) | 44(2.0) | 51(1.9) | 0.002 |
| 65-79 | 15(1.3) | 12(1.1) | 12(1.1) | 20(1.9) | 18(1.6) | 36(2.3) | 27(2.3) | 27(1.9) | 0.006 |
| ≥80 | 7(1.5) | 8(1.8) | 6(1.3) | 7(1.4) | 11(2.4) | 14(2.7) | 4(1.2) | 9(2.2) | 0.283 |
| Inotropics/vasopressors |  |  |  |  |  |  |  |  |  |
| 16-64 | 244(12.6) | 284(14.4) | 359(17.8) | 333(16.1) | 381(17.5) | 484(15.3) | 398(18.2) | 579(21.3) | ＜0.001 |
| 65-79 | 193(17.0) | 197(18.0) | 243(22.4) | 255(23.9) | 233(20.5) | 301(19.1) | 273(23.6) | 350(24.7) | ＜0.001 |
| ≥80 | 96(20.5) | 92(20.3) | 124(26.1) | 126(24.9) | 100(21.7) | 99(19.4) | 79(23.3) | 103(25.1) | 0.424 |
| Enteral nutrition support |  |  |  |  |  |  |  |  |  |
| 16-64 | 451(23.3) | 458(23.2) | 441(21.8) | 412(20.0) | 411(18.9) | 514(16.3) | 446(20.4) | 574(21.1) | ＜0.001 |
| 65-79 | 223(19.6) | 200(18.2) | 236(21.7) | 245(23.0) | 202(17.8) | 270(17.1) | 294(25.4) | 333(23.5) | 0.006 |
| ≥80 | 111(23.7) | 79(17.4) | 104(21.9) | 108(21.3) | 89(19.3) | 124(24.3) | 100(29.5) | 116(28.2) | 0.001 |
| **Length of stay(d), median(IQR)** |  |  |  |  |  |  |  |  |  |
| ICU LOS |  |  |  |  |  |  |  |  |  |
| 16-64 | 2.7(0.9-5.6) | 2.7(0.9-5.9) | 2.8(0.9-5.9) | 2.8(0.9-6.1) | 2.6(0.8-6.1) | 1.1(0.7-4.2) | 1.6(0.7-5.2) | 1.9(0.7-6.0) | 0.537 |
| 65-79 | 3.0(0.9-6.7) | 2.9(1.1-6.4) | 3.5(1.6-6.8) | 3.3(1.2-7.2) | 2.9(1.0-6.1) | 2.0(0.8-5.7) | 2.7(0.8-7.9) | 2.9(0.9-7.0) | 0.002 |
| ≥80 | 4.9(1.9-9.2) | 4.3(2.2-8.0) | 4.3(2.1-9.8) | 4.9(2.5-9.4) | 4.8(2.1-9.8) | 4.0(2.0-8.5) | 4.9(2.2-10.9) | 4.8(1.8-10.8) | 0.015 |
| Hospital LOS |  |  |  |  |  |  |  |  |  |
| 16-64 | 12.3(8.2-17.9) | 11.8(7.9-17.6) | 11.4(7.7-16.9) | 11.4(7.8-16.8) | 11.0(7.0-16.2) | 12.8(7.9-19.8) | 13.8(8.9-20.0) | 12.9(8.6-17.9) | ＜0.001 |
| 65-79 | 12.8(8.8-18.6) | 12.9(8.8-18.0) | 12.9(8.7-18.8) | 11.8(7.8-17.9) | 11.6(7.6-17.0) | 11.7(7.2-18.0) | 13.9(8.9-20.8) | 12.7(8.0-18.0) | 0.234 |
| ≥80 | 12.0(8.0-17.4) | 12.5(7.9-18.0) | 11.8(7.7-16.9) | 11.8(7.6-17.3) | 11.7(7.7-17.1) | 10.2(6.8-15.5) | 11.2(7.1-17.0) | 10.6(6.9-17.0) | 0.331 |

*Note:*P for change in ICU resource use over time.
