# Supplementary material for: Trends in admission, resource use and outcomes among elderly patients admitted to an intensive care unit in China
Source: PLoS One. 2026 May 15;21(5):e0348768. doi: 10.1371/journal.pone.0348768 (PMC13178899; doi:10.1371/journal.pone.0348768)
Supplement: S4 Table — (DOCX) [file pone.0348768.s004.docx]

**S4 Table.** Trends in Hospital Mortality.

|  | Year | | | | | | | |  |
| --- | --- | --- | --- | --- | --- | --- | --- | --- | --- |
|  | 2014 | 2015 | 2016 | 2017 | 2018 | 2019 | 2020 | 2021 | P |
| **Hospital mortality, n(%)** |  |  |  |  |  |  |  |  |  |
| 16-64 | 57(2.9) | 49(2.5) | 64(3.2) | 58(2.8) | 94(4.3) | 73(2.3) | 75(3.4) | 96(3.5) | 0.146 |
| 65-79 | 90(7.9) | 64(5.8) | 84(7.7) | 82(7.7) | 64(5.6) | 65(4.1) | 76(6.6) | 69(4.9) | ＜0.001 |
| ≥80 | 97(20.7) | 63(13.9) | 70(14.7) | 78(15.4) | 47(10.2) | 67(13.1) | 47(13.9) | 42(10.2) | ＜0.001 |
| Nonoperative, n(%) |  |  |  |  |  |  |  |  |  |
| 16-64 | 36(5.3) | 26(4.0) | 37(5.6) | 37(5.7) | 66(10.0) | 48(7.1) | 46(10.8) | 69(12.1) | ＜0.001 |
| 65-79 | 71(13.9) | 37(7.7) | 53(11.3) | 54(13.1) | 50(10.4) | 42(9.5) | 48(14.5) | 44(10.9) | 0.978 |
| ≥80 | 83(24.0) | 53(16.6) | 49(16.2) | 60(18.5) | 36(12.3) | 52(16.3) | 30(16.9) | 32(14.6) | 0.007 |
| Emergency surgical, n(%) |  |  |  |  |  |  |  |  |  |
| 16-64 | 14(4.1) | 14(3.6) | 17(3.3) | 14(2.9) | 20(3.5) | 19(2.7) | 15(2.7) | 24(3.4) | 0.389 |
| 65-79 | 8(5.7) | 15(10.6) | 17(9.6) | 18(7.9) | 7(3.3) | 11(3.1) | 20(7.1) | 20(6.1) | 0.085 |
| ≥80 | 4(10.8) | 6(15.0) | 11(18.0) | 11(12.4) | 7(9.9) | 10(10.4) | 11(13.3) | 8(12.1) | 0.588 |
| Elective surgical, n(%) |  |  |  |  |  |  |  |  |  |
| 16-64 | 7(0.8) | 9(1.0) | 10(1.2) | 7(0.8) | 8(0.9) | 6(0.3) | 14(1.2) | 3(0.2) | 0.006 |
| 65-79 | 11(2.2) | 12(2.5) | 14(3.2) | 10(2.4) | 7(1.6) | 12(1.6) | 8(1.5) | 5(0.7) | 0.003 |
| ≥80 | 10(11.8) | 4(4.2) | 10(9.0) | 7(7.4) | 4(4.1) | 5(5.3) | 6(7.6) | 2(1.6) | 0.019 |

*Note:*P for change in raw hospital mortality over time.
