# Supplementary material for: Trends in admission, resource use and outcomes among elderly patients admitted to an intensive care unit in China
Source: PLoS One. 2026 May 15;21(5):e0348768. doi: 10.1371/journal.pone.0348768 (PMC13178899; doi:10.1371/journal.pone.0348768)
Supplement: S5 Table — (DOCX) [file pone.0348768.s005.docx]

**S5 Table.** Multivariable logistic regression model for hospital mortality.

| Variable | Odds ratio(95% CI) | P |
| --- | --- | --- |
| **sex(male)** | 1.183(1.053–1.330) | 0.005 |
| **APS** | 1.186(1.176–1.197) | ＜0.001 |
| **Charlson comorbidity index** | 1.316(1.280–1.353) | ＜0.001 |
| **Admission type** |  |  |
| Nonoperative | 1 |  |
| Emergency surgical | 0.727(0.624–0.847) | ＜0.001 |
| Elective surgical | 0.387(0.320–0.468) | ＜0.001 |
| **Principal diagnosis** |  |  |
| Cardiovascular | 1 |  |
| Respiratory | 0.817(0.617–1.081) | 0.158 |
| Gastrointestinal | 1.015(0.798–1.293) | 0.898 |
| Hemotologic and oncologic | 0.438(0.342–0.561) | ＜0.001 |
| Infectious | 1.121(0.929–1.352) | 0.232 |
| Neurologic | 0.668(0.567–0.787) | ＜0.001 |
| Musculoskeletal and injuries | 0.923(0.670–1.270) | 0.624 |
| Other diseases | 0.488(0.331–0.718) | ＜0.001 |
| **ICU admission date(continuous-reported per year)** | **0.938(0.900**–**0.978)** | **0.003** |
| **Age group** |  |  |
| 16–64 years | 1 |  |
| 65–79 years | **2.633(1.956**–**3.543)** | ＜0.001 |
| ≥80 years | **4.711(3.433**–**6.464)** | ＜0.001 |
| **Interaction:** | |  |
| age 65–79 # ICU admission date | **0.921(0.870–0.976)** | **0.005** |
| age ≥80 # ICU admission date | **0.933(0.876–0.994)** | **0.033** |

Area under receiver operating characteristic curve = 0.887

Brier score = 0.039

Logistic regression analysis for in-hospital mortality (dependent variable);

independent variables: male sex, APS, Charlson comorbidity index, admission type, principal diagnosis, date/time of ICU admission entered into the logistic regression model as a continuous variable and reported as a change in odds of death per year, with an interaction term between date/time of ICU admission and the three age groups.
