# Supplementary material for: Trends in admission, resource use and outcomes among elderly patients admitted to an intensive care unit in China
Source: PLoS One. 2026 May 15;21(5):e0348768. doi: 10.1371/journal.pone.0348768 (PMC13178899; doi:10.1371/journal.pone.0348768)
Supplement: S6 Table — (DOCX) [file pone.0348768.s006.docx]

**S6 Table.** Summary of models examining adjusted hospital mortality over time, the interaction between age groups and time, and model performance characteristics.

| **Multivariable mixed effects logistic regression models** | | **P** |
| --- | --- | --- |
| **All patient types (all ages) – see supp. Table 1** | | |
| ICU admission date (reported as change per year), OR (95%CI) | 0.938(0.900–0.978) | 0.003 |
| Age group(age 16–64years as reference) | | |
| Age 65–79 years, OR(95%CI) | 2.633(1.956–3.543) | ＜0.001 |
| Age ≥80 years, OR(95%CI) | 4.711(3.433–6.464) | ＜0.001 |
| Interaction: age groups # ICU admission date(age 16–64years as reference) | | |
| Age 65–79 years # admission date, OR (95%CI) | 0.921(0.870–0.976) | 0.005 |
| Age ≥80 years # admission date, OR (95%CI) | 0.933(0.876–0.994) | 0.033 |
| Relative mortality risk-reduction per year (95%CI) | 6.1%(2.1–9.9%) | 0.003 |
| Area under receiver operating characteristic (brier score) | 0.887(0.039) |  |
| **All patient types (age 16-64 years)** | | |
| ICU admission date (reported as change per year), OR (95%CI) | 0.934(0.894–0.976) | 0.002 |
| Relative mortality risk-reduction per year (95%CI) | 6.1%(2.1–9.9%) | 0.003 |
| Area under receiver operating characteristic (brier score) | 0.901(0.041) |  |
| **All patient types (age 65-79 years)** | | |
| ICU admission date (reported as change per year), OR (95%CI) | 0.876(0.840–0.914) | ＜0.001 |
| Relative mortality risk-reduction per year (95%CI) | 13.5(9.8%–16.9%) | ＜0.001 |
| Area under receiver operating characteristic (brier score) | 0.857(0.040) |  |
| **All patient types (age ≥80 years)** | | |
| ICU admission date (reported as change per year), OR (95%CI) | 0.866(0.824–0.910) | ＜0.001 |
| Relative mortality risk-reduction per year (95%CI) | 12.3%(8.0%–16.5%) | ＜0.001 |
| Area under receiver operating characteristic (brier score) | 0.818(0.045) |  |

All models were adjusted for sex, APS, Charlson comorbidity index, admission type, principal diagnosis, date/time of ICU admission. OR = adjusted odds ratio; CI = confidence interval; ICU = Intensive Care Unit
