# Supplementary figures and images for: Trends in admission, resource use and outcomes among elderly patients admitted to an intensive care unit in China
Source: PLoS One. 2026 May 15;21(5):e0348768. doi: 10.1371/journal.pone.0348768 (PMC13178899; doi:10.1371/journal.pone.0348768)

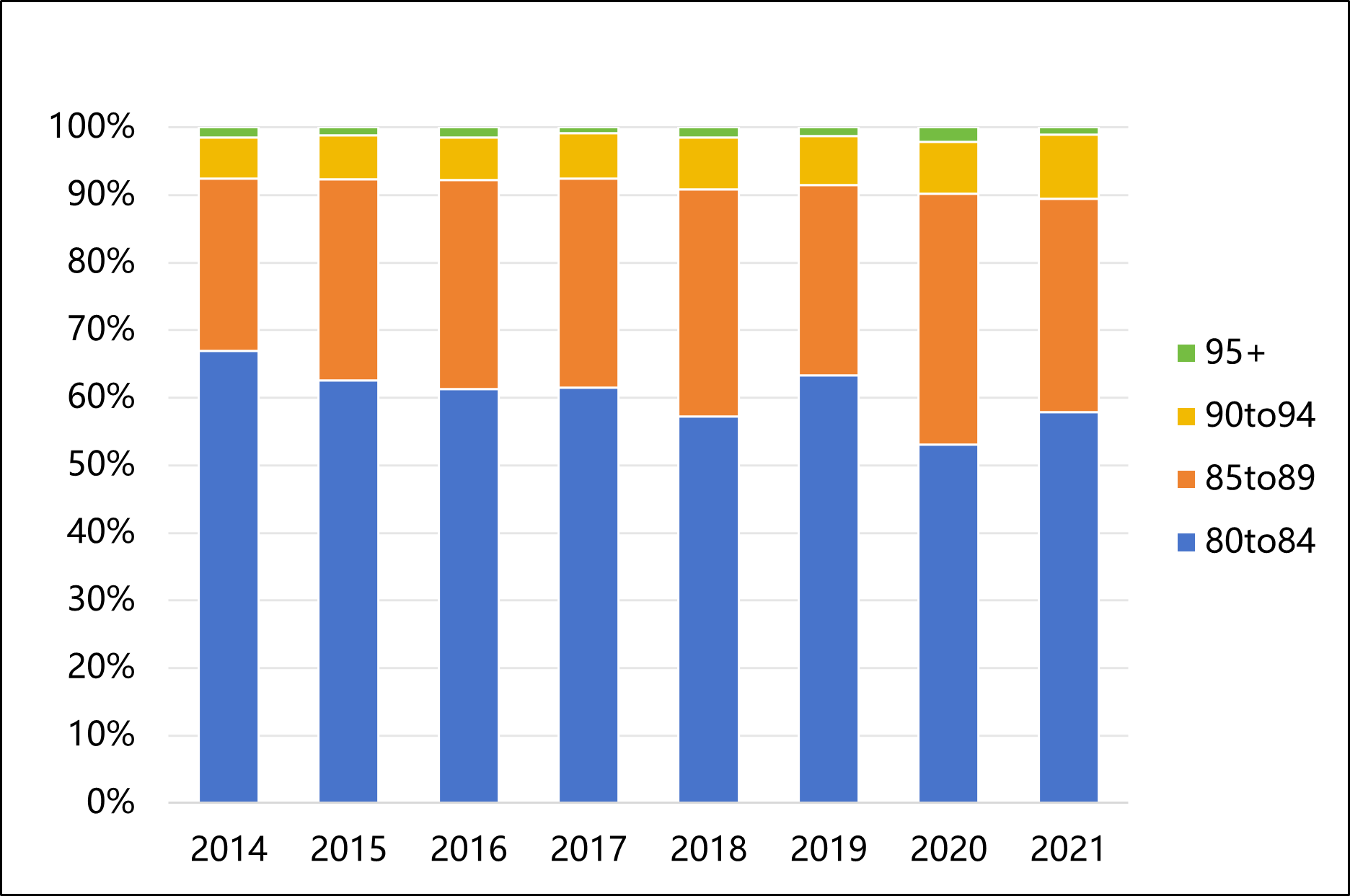

Supplement: S1 Fig — (TIF) [file pone.0348768.s007.tif]

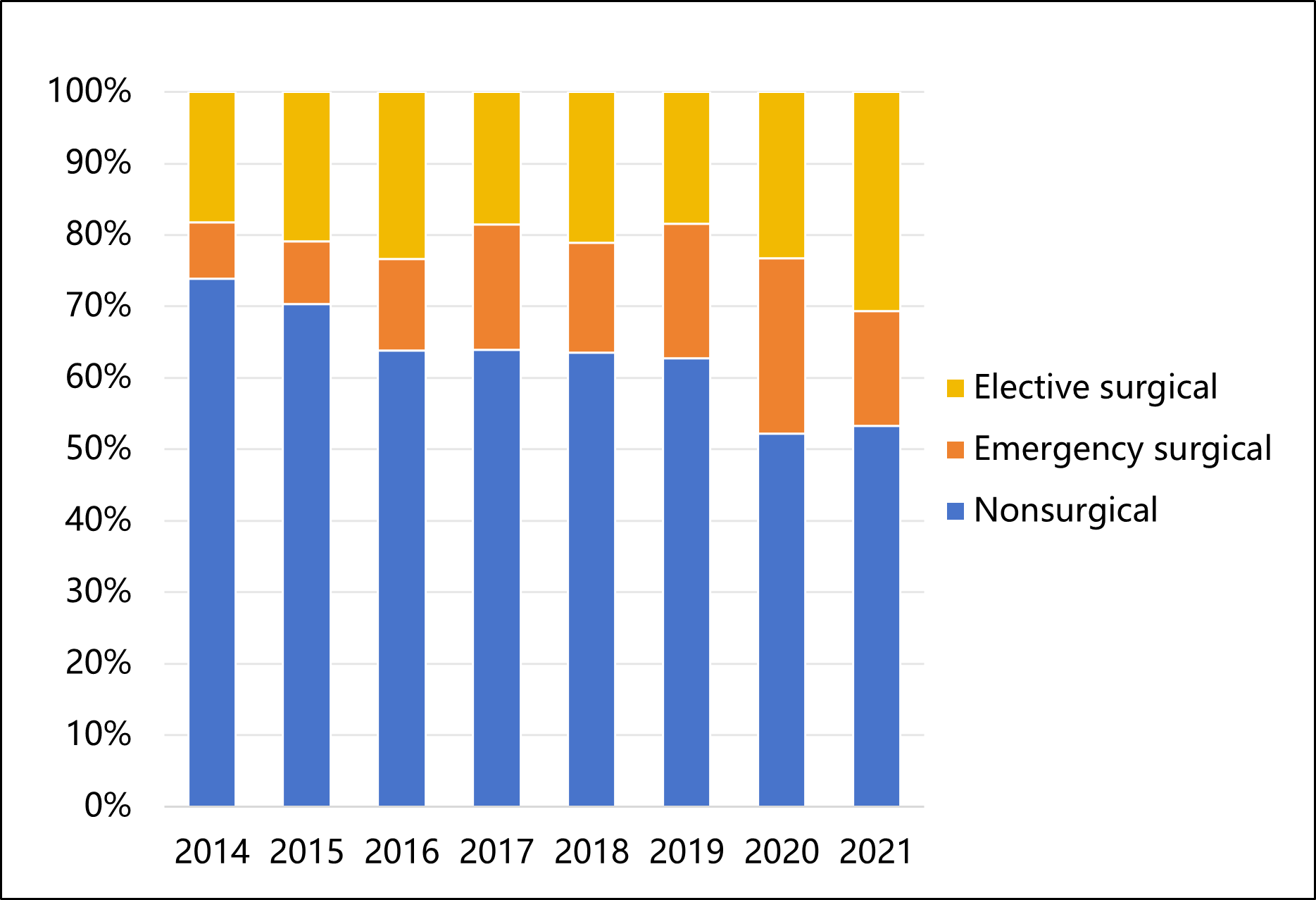

Supplement: S2 Fig — (TIF) [file pone.0348768.s008.tif]

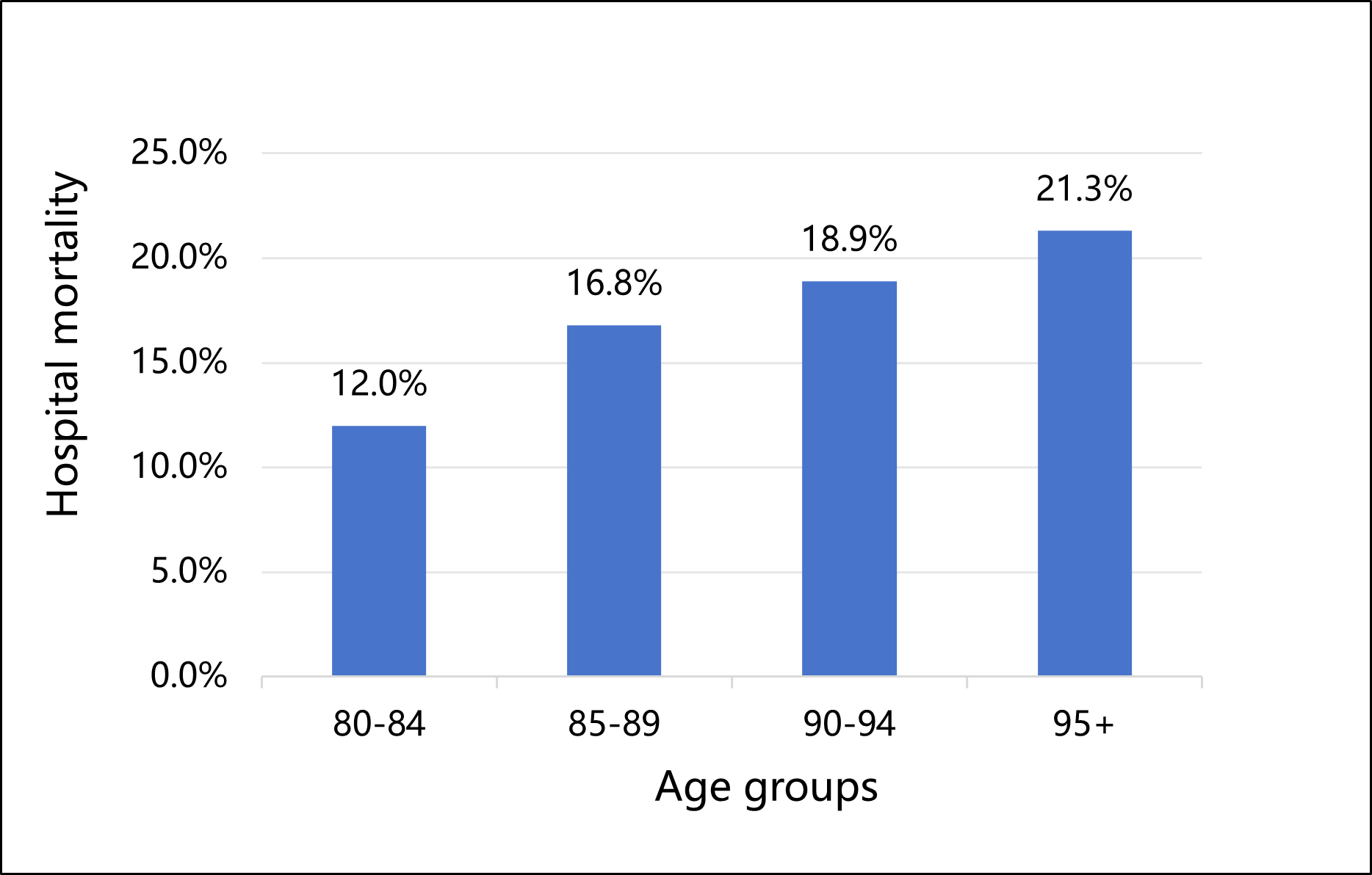

Supplement: S3 Fig — (TIF) [file pone.0348768.s009.tif]
