# Supplementary material for: Trends in admission, resource use and outcomes among elderly patients admitted to an intensive care unit in China
Source: PLoS One. 2026 May 15;21(5):e0348768. doi: 10.1371/journal.pone.0348768 (PMC13178899; doi:10.1371/journal.pone.0348768)
Supplement: S1 Text — (DOCX) [file pone.0348768.s010.docx]

SUPPLEMENTARY METHODS

Variables

We retrieved information on the number of test tubes collected for common laboratory analysis to estimate blood loss from repeated laboratory testing: arterial blood gas (1.0mL), biochemistry (2.5mL), complete blood count (2.0mL), blood culture (10mL), coagulation (volume 2.7mL)^1^.

1. Czempik, P.F., Wilczek, D., Herzyk, J., Krzych, Ł. J. Hospital-acquired anemia in patients hospitalized in the intensive care unit: a retrospective cohort study. *Journal of Clinical Medicine* **11**, 3939 (2022).
